# Immune cell counts and risks of respiratory infections among infants exposed pre- and postnatally to organochlorine compounds: a prospective study

**DOI:** 10.1186/1476-069X-7-62

**Published:** 2008-12-04

**Authors:** Anders Glynn, Ann Thuvander, Marie Aune, Anders Johannisson, Per Ola Darnerud, Gunnar Ronquist, Sven Cnattingius

**Affiliations:** 1 National Food Administration, Research and Development Department, PO Box 622, SE-751 26 Uppsala, Sweden; 2 Department of Anatomy and Physiology, Swedish University of Agricultural Sciences, PO Box 7070, SE- 750 07 Uppsala, Sweden; 3 Department of Medical Sciences, Clinical Chemistry, University Hospital of Uppsala, SE- 751 85, Uppsala, Sweden; 4 Department of Medical Epidemiology and Biostatistics, Karolinska Institutet, SE-171 77 Stockholm, Sweden; 5 The National Board of Health and Welfare, SE-106 30 Stockholm, Sweden

## Abstract

**Background:**

Early-life chemical exposure may influence immune system development, subsequently affecting child health. We investigated immunomodulatory potentials of polychlorinated biphenyls (PCBs) and *p,p'*-DDE in infants.

**Methods:**

Prenatal exposure to PCBs and *p,p'*-DDE was estimated from maternal serum concentrations during pregnancy. Postnatal exposure was calculated from concentrations of the compounds in mother's milk, total number of nursing days, and percentage of full nursing each week during the 3 month nursing period. Number and types of infections among infants were registered by the mothers (N = 190). White blood cell counts (N = 86) and lymphocyte subsets (N = 52) were analyzed in a subgroup of infants at 3 months of age.

**Results:**

Infants with the highest prenatal exposure to PCB congeners CB-28, CB-52 and CB-101 had an increased risk of respiratory infection during the study period. In contrast, the infection odds ratios (ORs) were highest among infants with the lowest prenatal mono-*ortho *PCB (CB-105, CB-118, CB-156, CB-167) and di-*ortho *PCB (CB-138, CB-153, CB-180) exposure, and postnatal mono- and di-*ortho *PCB, and *p,p'*-DDE exposure. Similar results were found for pre- and postnatal CB-153 exposure, a good marker for total PCB exposure. Altogether, a negative relationship was indicated between infections and total organochlorine compound exposure during the whole pre- and postnatal period. Prenatal exposure to CB-28, CB-52 and CB-101 was positively associated with numbers of lymphocytes and monocytes in infants 3 months after delivery. Prenatal exposure to *p,p'*-DDE was negatively associated with the percentage of eosinophils. No significant associations were found between PCB and *p,p'*-DDE exposure and numbers/percentages of lymphocyte subsets, after adjustment for potential confounders.

**Conclusion:**

This hypothesis generating study suggests that background exposure to PCBs and *p,p'*-DDE early in life modulate immune system development. Strong correlations between mono- and di-*ortho *PCBs, and *p,p'*-DDE exposures make it difficult to identify the most important contributor to the suggested immunomodulation, and to separate effects due to pre- and postnatal exposure. The suggested PCB and *p,p'*-DDE modulation of infection risks may have consequences for the health development during childhood, since respiratory infections early in life may be risk factors for asthma and middle ear infections.

## Background

Persistent and lipophilic organochlorine compounds, such as the industrial chemicals polychlorinated biphenyls (PCBs), the pesticide DDT, and dioxin-like contaminants polychlorinated dibenzo-*p*-dioxins (PCDDs) and dibenzofurans (PCDFs), are immunotoxic to animals and humans [1-7]. Suppression of the humoral and cellular immune system is one of the most sensitive endpoints after prenatal exposure of animals to the highly toxic dioxin-like compound 2,3,7,8-tetrachloro dibenzo-*p*-dioxin (TCDD) [6,7]. Children from Taiwan, who were accidentally exposed to high levels of both non-dioxin-like and dioxin like PCBs and polychlorinated dibenzofurans (PCDFs) prenatally, had higher rates of bronchitis, upper respiratory infections and middle ear infections than normally found in reference populations with low exposure [4,5].

A few studies have reported that exposures of the fetus and/or infant to background levels of non-dioxin-like and dioxin-like PCBs, the DDT metabolite *p,p'*-DDE, and PCDD/Fs are associated with alterations in markers of immune function, such as white blood cell (WBC) counts and numbers of lymphocyte subsets during childhood [8-13]. Diverging results have however been reported. On one hand no associations were found between WBC/lymphocyte subset counts and early life exposure to PCBs among Inuit infants [13]. On the other hand, among Dutch infants negative associations were found between monocyte counts and PCB exposure, and positive associations between CD8^+ ^cytotoxic T-cells and PCB exposure [12].

High prenatal PCB exposure has been associated with a decreased thymus size among neonates born in an area with high environmental load of both non-dioxin-like and dioxin-like PCBs in Eastern Slovakia [14]. Moreover, PCB exposure early in life has been found to be positively associated with acute *otitis media *and negatively associated with incidence of asthma or allergies later in life in children [10,11,15]. Studies of preschool and school children have also suggested that exposure to *p,p'*-DDE early in life may be a risk factor for acute otitis media and asthma later during childhood [13,16,17]. A decreased antibody response after tetanus and diphtheria vaccination was found among children exposed to high levels of PCBs early in life [18].

In order to better understand how PCB compounds and *p,p'*-DDE may influence children health, we studied the associations between pre- and postnatal PCB and *p,p'*-DDE exposure and numbers and percentages of WBCs and lymphocyte subsets in three month old infants. Moreover, associations between organochlorine compound exposure and upper/lower respiratory infections in infants were studied during the first three months of life. Compounds were assigned to one of four compound groups depending on biological activity and sources of exposure: non-dioxin-like tri- to pentachlorinated PCBs (CB-28, CB-52 and CB-101), non-dioxin-like di-*ortho *PCBs (CB-138, CB-153 and CB-180), dioxin-like mono-*ortho *PCBs (CB-105, CB-118, CB-156 and CB-167), and *p,p'*-DDE. Exposure to the di-*ortho *PCB congener CB-153 was used as a marker of total PCB exposure.

## Methods

### Study population and sampling

Between 1996 and 1999, 325 primiparous women, living and seeking prenatal care in Uppsala County, Sweden, agreed to donate a serum sample (82% participation rate) in late pregnancy (week 32–34) for chemical analysis of organochlorines [19]. PCB and DDE concentrations in serum lipids from this sampling were used as an estimate of prenatal PCB and DDE exposure of the infants, and 190 mother/child pairs had complete data for statistical analyses of associations between prenatal PCB and DDE exposure and diseases among the infants (Table 1).

**Table 1:** Characteristics of the participating mother/child pairs^a^

	Study groups								
Variable	Infections (N = 190)			White blood cells (N = 86)			Lymphocyte subsets (N = 52)		
Mother's age (yr)	28 (21–41)			29 (21–36)			29 (22–35)		
Infant's age (d)	92 (75–123)			93 (76–112)			93 (76–112)		

	Percent			Percent			Percent		

School yrs	≤ 13:50	14–16:24	>16:26	≤ 13:50	14–16:27	>16:23	≤ 13:43	14–16:30	>16:27
Smoking^b^	No:82	Yes:18		No:86	Yes:14		No:89	Yes:11	
Alcohol^b^	No:82	Yes:18		No:83	Yes:17		No:77	Yes:23	
Nursing^c^	Whole:81	Partial:19		Whole:84	Partial:16		Whole:91	Partial:9	
Infant's sex	Girl:44	Boy:56		Girl:58	Boy:42		Girl:57	Boy:43	
Vaccination	No:69	Yes:31		No:76	Yes:24		No:85	Yes:15	
Resp. infection	No:72	Yes:28		No:71	Yes:29		No:78	Yes:22	

^a^Mother's and infant's age: median (range). WBCs were analyzed in a subgroup of 86 infants, and within this subgroup lymphocyte subsets were analyzed among 52 infants with enough blood left after WBC count analysis.

^b^During pregnancy.

^c^Partial nursing also includes no nursing.

During the third week after delivery mothers from the group of 190 participants sampled milk while nursing their infants, using a manual breast pump and/or a passive mother's milk sampler [20]. The results of the PCB and DDE analyses of mother's milk was used in the assessment of postnatal exposure (see below) and 175 mother/infant pairs had complete data for statistical analyses of associations between postnatal PCB and DDE exposure and risk of infections.

Blood was sampled from infants 3 months after delivery for analysis of numbers and percentages of WBCs and lymphocyte subsets. The age of 3 months was chosen in order to facilitate comparison with other studies of immunomodulatory effects of early life PCB and DDE exposure [11,13]. Moreover, the majority of the infants in Sweden have mothers'milk as the only source of nutrition during the first 3 months of life, making confounding of results due to differences in sources of nutrition less pronounced. All the 325 mothers, donating blood in late pregnancy, were asked if they were willing to donate an infant blood sample after birth. Blood sampling could however not be continued during the whole study due to lack of financial resources. Therefore infants of mothers accepting blood sampling were sampled until 90 infants had been sampled. At that time point 277 mothers had been asked to donate a blood sample from their infants and the participation rate in the blood sampling of the infants was thus 32%. Among the 90 mother/infants pairs, data on numbers of WBCs were finally available from 81 infants and percentage data from 85 infants. Lymphocyte subsets could only be analyzed in 52 infants from this subgroup of infants, due to limited volumes of blood available. Among the infants with data on lymphocyte subsets, data on lymphocyte numbers and percentages were available for 47 and 52 infants, respectively. The study was approved by the Ethics Committee of the Medical Faculty at Uppsala University. All participating women gave their informed consent prior to the inclusion in the study group.

### Interviews and questionnaires

At 6–12 and 32–34 completed gestational weeks, in-person interviews regarding maternal characteristics were performed, using a structured questionnaire [19,21]. Data on maternal characteristics included age, height, body weight before pregnancy, body weight at interview, years of education, and alcohol consumption and smoking before and during pregnancy. Blood samples for cotinine analysis (used as indicator of smoking habits) were taken at both interview occasions.

After delivery, the mothers were visited by a midwife when the infant was 3 months old. In an in-person interview, using a structured questionnaire, the mothers answered questions about sex of the infant, vaccination, nursing habits, and infant health during the first 3 months. Women gave information about the extent of nursing for each week of the 3 month period (full nursing, partial nursing and no nursing). The participants also gave information about the health of the children each week from delivery to the date of the interview. It was asked if the infant had any infection or other disease during the 3 month period. If the answer was yes, the mother was asked to give information about type of disease (open ended question). The mother was asked to identify the week/weeks the infant had the disease in an almanac, and to give the number of days the symptoms lasted. It was also asked if the infant had a fever during these days, and number of days with a temperature above 39°C. Finally it was asked if the symptoms were treated by a physician and what type of treatment that was used. The interviewer and the mothers were blinded to the organochlorine compound body burdens of the mother.

### Organochlorine compound analysis

The lipid portion of serum samples was analyzed for the DDT metabolite *p,p'*-DDE, and 10 PCB congeners (IUPAC nos. 28, 52, 101, 105, 118, 138, 153, 156, 167 and 180). The analytical results were consequently lipid adjusted. Procedures for extraction, sample clean-up and analysis, and quality control are described in Glynn et al. [19]. In mother's milk the compounds were analyzed using a method and quality assurance described by Glynn et al. [20]. When concentrations were below the limit of quantification (LOQ) they were set to 50% of LOQ in the statistical analysis.

### Immune cell analysis

The hematological tests were performed at the Department of Clinical Chemistry, University Hospital of Uppsala. Capillary blood samples were collected in microtainer tubes (Sarstedt, Sweden). Differential counts were carried out by an automated instrument, Celldyn 4000 (Abbott Scandinavia AB, Solna, Sweden) based on a combination of optical characteristics and histochemical reactions. Number of total WBCs, and number and percentages of neutrophils, eosinophils, lymphocytes, and monocytes were recorded.

Analysis of lymphocyte subpopulations was performed on mononuclear cells prepared by Ficoll-Paque centrifugation and stained as described in Gräske et al. [22]. The following subpopulations were evaluated by flow cytometry: CD3^+ ^cells (T-lymphocytes), CD19^+ ^cells (B-lymphocytes), CD4^+ ^cells (T-helper cells), CD8^+ ^cells (cytotoxic cells), and CD56^+ ^cells (NK-cells).

### Calculations and statistics

Lipid-adjusted serum organochlorine compound concentrations among the mothers in late pregnancy were used as a measure of prenatal exposure of the fetus [23-25]. Postnatal exposure (PE) (ng or pg/g*days) was calculated from the organochlorine concentration (ng or pg/g fresh weight) in mother's milk three weeks after delivery (Oconc), the number of days of nursing (Nd), and the percentage of full nursing during the study period (%N) using the equation

PE (ng or pg/g*days) = Oconc*Nd*(%N/100).

For women who did not nurse their infants PE = 0. The nursed infant's cumulative intake of organochlorine compounds is the product of the concentration of the organochlorine compound in question in the mothers' milk (per gram fresh weight), the amount of milk consumed per day (in grams), and the number of days of nursing [26,27]. We did not have information about the amount of milk consumed by the infants. Instead we had information about the degree of nursing each week (full, partial or none). Consequently, the degree of nursing was used instead of the amount of mothers' milk consumed in the intake calculation.

In the exposure analysis, tri- to penta-chlorinated CB-28, CB-52 and CB-101, with no dioxin-like biological activity, were grouped together (CB 28+52+101) because serum levels of these compounds showed low correlations with serum levels of the other organochlorine compounds (Spearman's r = 0.13–0.24). These three congeners have been detected in elevated levels in indoor air of buildings containing PCB-laden building materials and in the blood of residents of such buildings [28-31]. CB-138, CB-153 and CB-180 (di-*ortho *PCBs) were grouped together because of high correlations between mother's serum concentrations of these congeners (r > 0.90), and because of their lack of dioxin-like biological activity [32]. Dioxin-like mono-*ortho *PCBs consisted of CB-105, CB-118, CB-156 and CB-167 [32]. *p,p'*-DDE was treated separately in the statistical analysis. In order to improve the possibilities to compare our PCB results with those of other similar studies, statistical analysis was also performed using exposure to the di-*ortho *PCB congener CB-153 as independent exposure variable. Concentrations of CB-153 in blood serum of the mothers was highly correlated with concentrations of total PCB (Spearman's r = 0.97), showing that CB-153 exposure is a good marker of total PCB exposure.

Exposure to dioxin-like mono-*ortho *PCBs were summarized using WHO 1998 toxic equivalency factors (TEFs) for the congeners CB-105 (TEF = 0.0001), CB-118 (0.0001), CB-156 (0.0005), and CB-167 (0.00001) [32]. The concentration of the individual congeners was multiplied with the TEF of the congener in question [32], and the resulting concentration of toxicity equivalents (TEQs) for each congener was then summarized in to a sum of mono-*ortho *TEQ concentration for each sample.

Statistical analysis was performed using MINITAB^® ^For Windows, 14. Spearman's rank correlation analysis was used in analysis of correlations between different exposure variables. The associations between immune cell numbers/percentages and organochlorine compound exposure were explored by linear regression analysis. Six infants had an ongoing infection at the time of sampling, or an infection within a period of 7 days before the sampling day. These infants were not included in the statistical analyses. Regression analysis was performed on logarithmically transformed organochlorine compound exposure data, since the distributions of data closely followed a log-normal distribution. However, in the case of prenatal exposure to CB 28+52+101 many women had serum levels below LOQ and this exposure variable was therefore categorized. Some women did not nurse their infants at all, and postnatal exposure of these infants was set to zero. This made logarithmic transformation impossible and postnatal exposure was therefore categorized. Prenatal CB 28+52+101 exposure was categorized in 3 categories. The study participants could not be categorized in tertiles since over 40% had concentrations below the LOQ. Mother/infants pairs with CB 28+52+101 concentrations below the LOQ were grouped in the reference category and the rest of the study participants divided up in equal numbers in the two other categories depending on exposure levels. Postnatal organochlorine compound exposure was categorized in tertiles when possible, otherwise an effort was made to have equal numbers of study participants in each of the three exposure categories.

In the statistical analysis of immune cell results the significance level was set to p ≤ 0.01, since multiple comparisons were made. In cases when a statistically significant association between immune cells and organochlorine compound exposure was found in simple regression analysis, multiple regression analysis was used to adjust the associations for potential confounders. We included lifestyle/medical factors in the multiple regression analyses that have previously been reported to be associated with increased or decreased risk of immune-related diseases [33-38]. Potential confounders included in the analysis were age of the mother, smoking during pregnancy (non-smoker/former smoker/smoked during pregnancy), alcohol consumption during pregnancy (no/yes), mother's education (≤ 13 years of education/14–16 years of eduction/>16 years of eduction), vaccination status of the infant at the time of sampling (no/yes), nursing of the infant (no or partial nursing/full nursing), age of the infant, and infant's past history of respiratory infections during the study period (up to 7 days before sampling, no/yes). In linear regression analysis a few observations with a standardized residual ≥ 3.0 were omitted from the data sets due to a large impact on the regression results.

In the analysis of infection results (no/yes), odds ratios (ORs) and 95% confidence intervals (CIs) were calculated using logistic regression. Respiratory infection (influenza-like symptoms, common cold, or common cold with cough) (N = 54) was the only health problem that was frequent enough to allow statistical analysis. Other health problems reported were stomach problems (N = 1), navel infection (N = 1), cows' milk intolerance/allergy (N = 1), urinary infection (N = 1), eye infection (N = 2) and middle ear infection (N = 2). In the logistic regression analysis all independent variables were categorized in order to handle outlier problems. First, ORs were calculated in regressions with the exposure to each organochlorine compound group as the only independent variable. In the next step the ORs were adjusted for the same possible confounders as in the analyses of immune cell counts/percentages (except infant's history of infection, see above).

A stricter definition of respiratory infection was also used (three days or more of infection, N = 50) in the statistical analysis. The information about number of infections during the study period could not be used in the statistical analyses of the results, since only 7 infants had more than one infection during the study period. Similarly, too few infants had been examined by a physician. It was not possible to use the information about infection in combination with information about body temperature in the statistical analysis, since there were too many missing values for the latter variable.

We also analyzed the associations between respiratory infections and the total exposure to PCBs and *p,p'*-DDE during the whole prenatal and postnatal period. This analysis was based on a summation of each infant's categorized pre-and postnatal exposure to all compound groups. We did not calculate a sum of the absolute concentrations since such a summation assumes equal immunomodulatory potency of all the studied compounds. For example, when absolute concentrations are summed up, effects of relatively potent compounds present at low concentrations may be underestimated, and effects of less potent compounds present at high concentrations overestimated. We avoided this by summing up exposure scores for each compound group. First each infant's pre- and postnatal PCB and *p,p'*-DDE exposures were separately scored, with the lowest score 1 given to the lowest exposure quartile and the highest score 4 given to the highest exposure quartile (scores 1–3 for prenatal CB 28+52+101 exposure). The scores for pre- and postnatal exposures were then summarized for each compound group. For example, if an infant scored 1 for prenatal exposure to *p,p'*-DDE and scored 3 for postnatal *p,p'*-DDE exposure then the infant was assigned a score of 4 in the summation of pre- and postnatal *p,p'*-DDE exposure. The resulting sum of the pre-and postnatal exposure scores of each compound group was then added to get the total exposure score. In this case, if an infant had scored 5 for total CB 28+52+101 exposure, 8 for mono-*ortho *PCB exposure, 6 for di-*ortho *PCB exposure, and 5 for *p,p'*-DDE exposure, the total organochlorine compound exposure score was summed up to 24. The total exposure scores were subsequently divided into quartiles in the final statistical analyses of associations between infections and total organochlorine exposure. The level of statistical significance in logistic regression analysis was set to p ≤ 0.05.

## Results

### Personal characteristics

Table 1 gives the personal characteristics of the whole study group (infection), the subgroup of mother/infant pairs that donated blood for WBC counts, and mother/infant pairs within this subgroup that donated enough blood for lymphocyte subset analyses. A comparison of personal characteristics between the study participants that did not donate infant blood (N = 104) with those donating infant blood (N = 86) showed no significant differences in age of the mothers and infants (t-test, p = 0.29 and p = 0.82) (Table 1). No difference was found between the two subgroups in smoking during pregnancy (Chi-square test, p = 0.064), alcohol consumption during pregnancy (Chi-square test, p = 0.89), years of education (Chi-square test, p = 0.57), nursing (Chi-square test, p = 0.28), and respiratory infections (Chi-square test, p = 0.52). Furthermore, pre- and postnatal organochlorine compound exposure did not differ between the groups (t-test, p = 0.054–0.84). However, there were more girls than boys in the subgroup with blood samples, whereas the reverse was evident among mother/infant pairs that did not donate infant blood (Chi-square test, p = 0.002). Fewer had been vaccinated at the end of the study period among infants with blood samples (Chi-square test, p = 0.038).

### Pre- and postnatal exposure

The median prenatal exposure of the sum of CB-28, CB-52 and CB-101 was low (Table 2). Over 40% of the mothers in the whole study group had concentrations of all the three substances in serum lipids below the LOQ. Concentrations ≥ 10 ng/g lipid were however found in 15% of the study participants. Median concentrations of di-*ortho *PCBs (CB-138, CB-153 and CB-180) and *p,p'*-DDE were similar, and the concentrations varied 8- to 26-fold. The concentrations of mono-*ortho *PCB TEQs varied 9-fold or more (Table 2).

**Table 2:** Organochlorine concentrations in mother's serum lipids in late pregnancy and infant exposure from mother's milk^a^

Exposure	Study groups		
Serum^b^	Infections	White blood cells	Lymphocyte subsets
CB 28+52+101	4 (3–427)	4 (3–427)	4 (3–19)
CB-153	60 (23–179)	57 (23–158)	59 (23–158)
Di-*ortho *PCB	131 (44–362)	127 (44–342)	129 (44–342)
Mono-*ortho *PCB TEQ	4 (1–20)	4 (1–11)	4 (1–9)
*p,p'*-DDE	88 (21–622)	85 (24–622)	83 (29–622)

Mother's milk exposure^c^			

CB 28+52+101	8 (0–155)	8 (0–76)	9 (0–39)
CB-153	187 (0–853)	174 (0–396)	186 (0–396)
Di-*ortho *PCB	364 (0–1666)	351 (0–830)	372 (0–830)
Mono-*ortho *PCB TEQ	11 (0–54)	11 (0–26)	11 (0–26)
*p,p'*-DDE	311 (0–2199)	289 (0–2199)	306 (0–2199)

^a^Median (range). Di-*ortho *PCB = CB-138, CB-153, CB-180; Mono-*ortho *PCB TEQ = CB-105, CB-118, CB-156, CB-167 [32]. The study group "Infections" consists of all study participants (prenatal exposure N = 190; postnatal exposure N = 175), the "White blood cells" group is a subgroup of infants among the study participants which had their WBC counts analyzed (N = 86), and within this subgroup lymphocyte subsets were analyzed in the "Lymphocyte" group composed of infants with enough blood left after WBC count analysis (N = 52).

^b^ng/g lipid. Mono-*ortho *PCBs: pg TEQ/g lipid [32]. Serum sampled week 32–34.

^c^ng/g fresh weight*days. Mono-*ortho *PCBs: pg TEQ/g fresh weight*days. Calculated as organochlorine concentration in mother's milk on a fresh weight basis*days of nursing*(%of full nursing/100).

Correlations between serum concentrations of CB 28+52+101 and the other organochlorines in the total study group (infection) were weak, with Spearman correlation coefficients ranging from 0.13 to 0.24. Concentrations of CB-153, di-*ortho *PCBs and mono-*ortho *PCB TEQs were strongly correlated (r ≥ 0.93), whereas correlations between these PCBs and *p,p'*-DDE were less strong (r = 0.66–0.75).

Variation in organochlorine exposure from mother's milk during the first 3 months of infancy was large (Table 2). The correlation between postnatal exposure to CB-153, di-*ortho *PCBs and mono-*ortho *TEQs was strong (r ≥ 0.93). Other correlations between the studied compound groups were less strong (r = 0.40–0.82). Pre- and postnatal exposure of each compound group was significantly correlated, ranging from r = 0.71 to r = 0.78.

### Organochlorine compound exposure and WBCs

**Figure 1:**
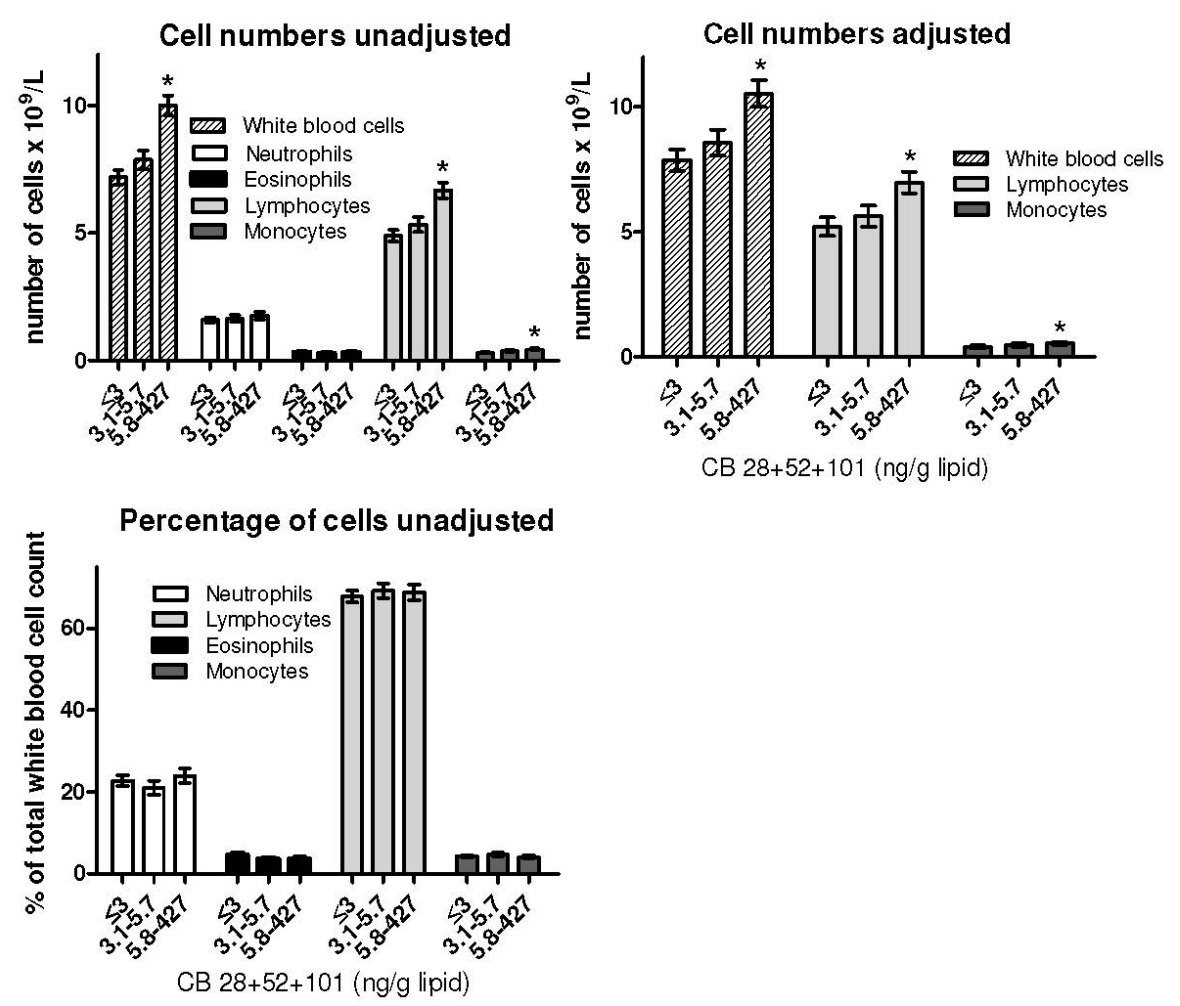
**White blood cells (WBC) and prenatal exposure to PCB congeners CB-28, CB-52 and CB-101**. Unadjusted and adjusted means (± SE) of WBC numbers and percentages in three months old infants prenatally exposed to the sum of CB-28, CB-52 and CB-101. Lipid adjusted serum concentrations of the PCB compounds in the blood of the mothers in late pregnancy (week 32–34) were used as a measure of prenatal exposure. Infants having an infection within a 7 day period before sampling were excluded from the statistical analyses. Adjusted means were calculated in cases when statistically significant results were found in the unadjusted analysis. Results were adjusted for age of the mother, smoking and alcohol during pregnancy, mother's education, vaccination of the infant, nursing of the infant, age of the infant, and infant's history of respiratory infections. The exposure variable was categorized since over 40% of the mothers had serum lipid levels of the PCBs below the limit of quantification (reference category 1). An effort was made to have equal numbers of participants in the two other exposure categories. *Significantly different from the group with the lowest exposure (reference category 1) (N = 75–79, p ≤ 0.01).

The numbers and percentages of WBCs are presented in Table 3. In both simple and multivariate regression analysis, infants with the highest CB 28+52+101 exposure had significantly higher mean numbers of total WBCs, lymphocytes and monocytes than infants in the reference category with the lowest exposure (Figure 1). The percentage of eosinophils was negatively associated with prenatal *p,p'*-DDE exposure (Table 4). Otherwise, no significant associations between prenatal exposure and WBC counts/percentages were found (Figure 1, Table 4).

**Table 3:** Numbers and percentages of white blood cells (WBC) and lymphocyte subsets in 3 month old infants^a^

Differential count	N	No. of cells × 10^9^/L	N	% of WBC
White blood cells	81	8 (5–15)		
Neutrophils	80	1.6 (0.6–5.7)	85	21 (8–47)
Eosinophils	80	0.3 (0.1–1.0)	85	4 (0.5–10)
Lymphocytes	80	5.4 (2.9–9.5)	85	70 (37–86)
Monocytes	80	0.3 (0.1–1.4)	85	4 (1–15)

Lymphocyte subsets				% of lymphocytes

CD19^+^	47	0.8 (0.1–2.0)	51	16 (2–34)
CD3^+^	47	3.7 (1.5–7.3)	52	70 (38–85)
CD4^+^CD8^-^	47	3.1 (1.0–5.3)	52	55 (24–72)
CD4^-^CD8^+^	47	0.7 (0.2–2.1)	52	13 (5–23)
CD56^+^	47	0.05 (0–0.1)	52	0.8 (0–2.2)

^a^Median (range)

**Table 4:** Regression coefficients for associations between organochlorine exposure prenatally and numbers and percentages of white blood cells and lymphocyte subsets^a^

	CB 153	Di-*ortho *PCB	Mono-*ortho *PCB TEQ	*p,p'*-DDE
White blood cell count^b^	0.01 ± 0.52	0.04 ± 0.54	0.31 ± 0.45	0.46 ± 0.36
Neutrophil numbers^b^	0.04 ± 0.18	0.05 ± 0.18	0.08 ± 0.15	-0.03 ± 0.12
Neutrophil %^b^	1.11 ± 2.27	1.09 ± 2.23	0.36 ± 1.85	-0.80 ± 1.51
Eosinophil numbers^b^	-0.00 ± 0.04	0.001 ± 0.04	0.01 ± 0.03	-0.06 ± 0.03
Eosinophil %^b^	0.05 ± 0.57	0.02 ± 0.59	0.002 ± 0.49	-1.06 ± 0.38*
Eosinophil %^c^				-1.62 ± 0.46*
Lymphocyte numbers^b^	-0.03 ± 0.41	-0.01 ± 0.43	0.17 ± 0.35	0.48 ± 0.28
Lymphocyte %^b^	-1.05 ± 2.31	-1.07 ± 2.39	0.06 ± 1.99	1.41 ± 1.61
Monocyte numbers^b^	0.004 ± 0.05	0.01 ± 0.05	-0.01 ± 0.04	0.04 ± 0.03
Monocyte %^b^	0.24 ± 0.51	0.32 ± 0.53	-0.15 ± 0.44	0.36 ± 0.36

CD19^+ ^numbers^b^	-0.07 ± 0.16	-0.06 ± 0.16	-0.07 ± 0.15	0.03 ± 0.11
CD19^+ ^%^b^	-0.41 ± 2.55	0.05 ± 2.6	0.87 ± 2.3	-0.81 ± 1.7
CD3^+ ^numbers^b^	-0.08 ± 0.46	-0.05 ± 0.47	-0.19 ± 0.42	0.42 ± 0.30
CD3^+ ^%^b^	-3.33 ± 4.09	-2.9 ± 4.2	-3.1 ± 3.8	-0.89 ± 2.8
CD4^+^CD8^- ^numbers^b^	0.04 ± 0.36	0.13 ± 0.37	0.05 ± 0.33	0.29 ± 0.24
CD4^+^CD8^- ^%^b^	-0.18 ± 3.89	0.17 ± 4.02	0.03 ± 3.68	-0.63 ± 2.70
CD4^-^CD8^+ ^numbers^b^	-0.22 ± 0.08	-0.24 ± 0.09	-0.19 ± 0.08	-0.15 ± 0.06
CD4^-^CD8^+ ^%^b^	-3.54 ± 1.19*	-3.84 ± 1.23*	-3.28 ± 1.12*	-1.98 ± 0.92
CD4^-^CD8^+ ^%^c^	-3.69 ± 1.93	-4.08 ± 2.05	-4.91 ± 2.05	
CD56^+ ^numbers^b^	0.01 ± 0.01	0.01 ± 0.01	0.003 ± 0.009	0.003 ± 0.007
CD56^+ ^%^b^	0.27 ± 0.18	0.24 ± 0.19	0.18 ± 0.17	0.07 ± 0.13

^a^Infants with an ongoing infection at the time of sampling were excluded, as well as infants that had an infection within 7 days before sampling. Prenatal exposure: lipid adjusted mother's serum organochlorine compound concentrations in late pregnancy (week 32–34). Di-*ortho *PCB = CB-138, CB-153, CB-180; Mono-*ortho *PCB TEQ = CB-105, CB-118, CB-156, CB-167 [32]. White blood cell counts: N = 75–79. Lymphocyte subsets: N = 45–50.

^b^Regression coefficients from simple regression analysis (mean ± SE)

^c^Partial regression coefficients (mean ± SE) adjusted for age of the mother, smoking and alcohol during pregnancy, mother's education, vaccination of the infant, nursing of the infant, age of the infant, and infant's history of respiratory infections.

*p ≤ 0.01.

Numbers and percentages of WBCs were not significantly associated with postnatal exposure to any of the analyzed organochlorines, except for a higher percentage of lymphocytes among infant with the highest post-natal *p,p'*-DDE exposure in the univariate analysis (see Additional file 1 and Additional file 2).

### Organochlorine exposure and lymphocyte subsets

**Figure 2:**
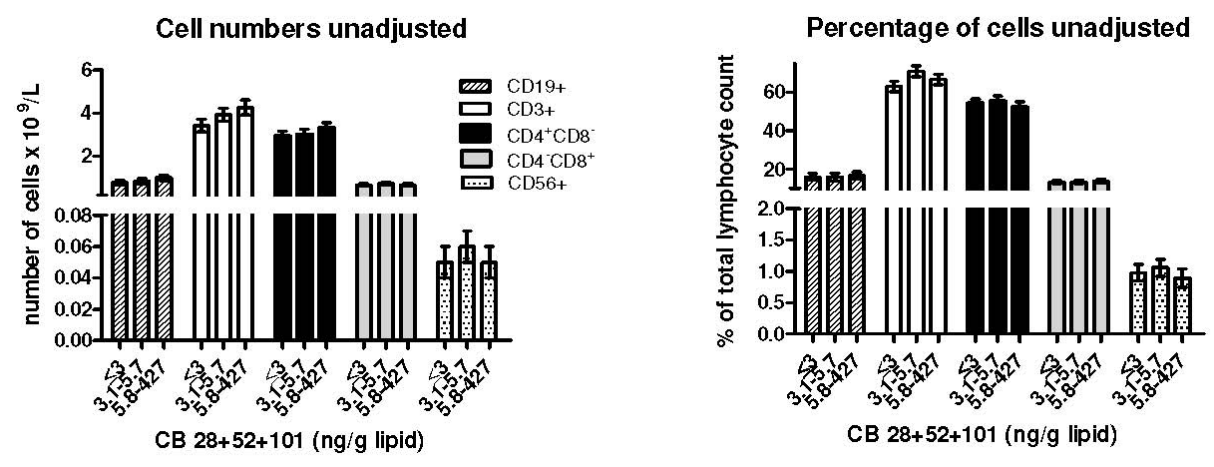
**Lymphocyte subsets and prenatal exposure to the PCB congeners CB-28, CB-52 and CB-101**. Unadjusted means (± SE) of numbers and percentages of lymphocyte subsets in three months old infants prenatally exposed to the sum of CB-28, CB-52 and CB-101. Lipid-adjusted serum concentrations of the PCB compounds in the blood of the mothers in late pregnancy (week 32–34) were used as a measure of prenatal exposure. Infants having an infection within a 7 day period before sampling were excluded from the statistical analyses. The exposure variable was categorized since over 40% of the mothers had serum lipid levels of the PCBs below the limit of quantification (reference category 1). An effort was made to have equal numbers of participants in the two other exposure categories. *Significantly different from the group with the lowest exposure (reference category 1) (N = 45–50, p ≤ 0.01).

We found a statistically significant negative association between percentage of CD4^-^CD8^+ ^cells and prenatal exposure to CB-153, di-*ortho *and mono-*ortho *PCBs in the univariate analysis (Table 4). After adjustment for potential confounders the associations were not statistically significant. In other cases, numbers and percentages of different lymphocyte subsets were not associated with prenatal exposure (Figure 2, Table 4). Postnatal exposure of the infants to the organochlorines was not significantly associated with numbers and percentages of lymphocyte subsets, except for a significantly higher number and percentage of CD56^+ ^among infants in the second highest exposure group of post-natal CB-153 exposure (see Additional file 3 and Additional file 4).

### Respiratory infections

Infants with the highest prenatal CB 28+52+101 exposure had a significantly increased odds ratio for respiratory infections during the first three months after birth compared with the lowest exposed infants (Table 5). This finding did not change in multivariate-adjusted logistic regression analysis. Moreover, a lowered odds ratio was found for infants in the second to fourth exposure category of prenatal di-*ortho *PCB exposure, although the difference between the highest exposure category and the reference category only showed borderline statistical significance (Table 5). Similar results were found for prenatal CB-153 exposure. A lowered odds ratio was also found for infants in the highest exposure category of prenatal mono-*ortho *PCB exposure in the multivariate-adjusted analysis (Table 5). Infants with the lowest prenatal *p,p'*-DDE exposure had a higher odds ratio than infants with higher *p,p'*-DDE exposures, but the difference never reached statistical significance. Similar results were evident when a stricter definition of respiratory infection (3 days or more of infection) was used (Additional file 5).

**Table 5:** Odds ratios (95% CI) for associations between respiratory infection during the first 3 months after birth and pre- or postnatal exposure to organochlorine compounds^a^

	Model^b^	Exposure categories^c^			
**Prenatal**		Category 1	Category 2	Category 3	Category 4
CB 28+52+101	Unadjusted	1.0	1.5 (0.66–3.3)	2.6 (1.2–5.6)*	
	Multivariate	1.0	1.7 (0.71–4.1)	3.4 (1.4–7.8)*	
CB 153	Unadjusted	1.0	0.43 (0.18–1.04)	0.37 (0.15–0.91)*	0.54 (0.23–1.3)
	Multivariate	1.0	0.45 (0.17–1.19)	0.30 (0.10–0.88)*	0.42 (0.12–1.4)
Di-*ortho *PCB	Unadjusted	1.0	0.35 (0.14–0.86)*	0.34 (0.14–0.83)*	0.49 (0.21–1.2)
	Multivariate	1.0	0.28 (0.10–0.79)*	0.23 (0.07–0.71)*	0.29 (0.08–1.0)
Mono-*ortho *PCB TEQ	Unadjusted	1.0	0.71 (0.30–1.7)	0.54 (0.23–1.3)	0.46 (0.19–1.2)
	Multivariate	1.0	0.58 (0.22–1.6)	0.34 (0.10–1.1)	0.23 (0.06–0.91)*
*p,p'*-DDE	Unadjusted	1.0	0.67 (0.27–1.6)	0.73 (0.30–1.78)	0.80 (0.34–1.9)
	Multivariate	1.0	0.64 (0.24–1.7)	0.69 (0.25–1.9)	0.74 (0.25–2.2)

**Postnatal**					

CB 28+52+101	Unadjusted	1.0	0.51 (0.19–1.4)	0.72 (0.28–1.9)	1.4 (0.58–3.4)
	Multivariate	1.0	0.44 (0.14–1.4)	0.65 (0.23–1.8)	1.4 (0.53–3.8)
CB 153	Unadjusted	1.0	0.55 (0.22–1.4)	0.66 (0.27–1.6)	0.37 (0.14–0.97)*
	Multivariate	1.0	0.38 (0.12–1.2)	0.40 (0.13–1.2)	0.20 (0.06–0.73)*
Di-*ortho *PCB	Unadjusted	1.0	0.48 (0.19–1.2)	0.53 (0.21–1.3)	0.31 (0.12–0.83)*
	Multivariate	1.0	0.27 (0.08–0.87)*	0.26 (0.08–0.85)*	0.14 (0.04–0.50)*
Mono-*ortho *PCB TEQ	Unadjusted	1.0	0.36 (0.14–0.97)*	0.53 (0.21–1.3)	0.69 (0.29–1.7)
	Multivariate	1.0	0.23 (0.07–0.79)*	0.37 (0.12–1.2)	0.33 (0.10–1.1)
*p,p'*-DDE	Unadjusted	1.0	0.25 (0.09–0.71)*	0.53 (0.21–1.3)	0.69 (0.29–1.7)
	Multivariate	1.0	0.18 (0.06–0.60)*	0.40 (0.14–1.2)	0.52 (0.17–1.5)

^a^Prenatal exposure: lipid adjusted mother's serum organochlorine compound concentrations in late pregnancy (week 32–34). Postnatal exposure: mother's milk concentrations on a fresh weight basis*days of nursing*(%of full nursing/100). Di-*ortho *PCB = CB-138, CB-153, CB-180; Mono-*ortho *PCB TEQ = CB-105, CB-118, CB-156, CB-167 [32]. Prenatal exposure:N = 190. Postnatal exposure:N = 175.

^b^Multivariate model also included the independent variables age of the mother, smoking and alcohol during pregnancy, mother's education, vaccination of the infant, nursing of the infant, and age of the infant.

^c^Pre- and postnatal exposure was categorized with the lowest exposure in Category 1 and increasing exposure with increasing Category number. Efforts were made to have equal numbers of participants in the exposure categories for each compound group.

*Significantly different from the odds ratio of the reference category (p ≤ 0.05).

In the analysis of postnatal exposure, the OR for respiratory infections among infants in the highest exposure category of CB-153 and di-*ortho *PCB exposure was significantly lower than that of the reference category in the unadjusted analysis (Table 5). Multivariate analysis did not change this difference markedly. Significantly lowered ORs were observed for the second exposure category of *p,p'*-DDE and mono-*ortho *PCB, suggesting a U-shaped relationship (Table 5). An U-shaped relationship was also suggested for CB 28+52+101, but the differences in odds ratio between the reference category and the other exposure categories were not statistically significant. Similar results were found when a stricter definition of respiratory infection was used (infection for 3 or more days) was used in the statistical analyses (Additional file 5).

ORs for respiratory infections among infants in the second, third and fourth categories of total organochlorine compound exposure during the whole pre- and postnatal period were 0.74 (95% confidence interval:0.29–1.88), 0.36 (0.13–0.96), and 0.68 (0.28–1.68), respectively.

## Discussion

In this exploratory study of associations between early life exposure to organochlorine compounds and risks of respiratory infections among 3 month old infants, diverging associations between exposure to organochlorines compounds and infection risk were found. Our results suggest that high prenatal background exposure to the PCB congeners CB-28, CB-52 and CB-101 may increase the risk of respiratory infections among infants. The reverse was suggested among infants with high background exposure to mono- and di-*ortho *PCBs prenatally, and high mono- and di-*ortho *PCB and *p,p'*-DDE exposure postnatally, although the dose-response in some cases was not monotonic. Similar results were found for the single di-*ortho *congener CB-153, which is a good marker for total PCB exposure. The strong correlations between dioxin-like mono-*ortho *PCB, non-dioxin-like di-*ortho *PCB (including CB-153), and *p,p'*-DDE exposures among the Uppsala infants make it difficult to suggest which of the organochlorine compound groups that contributes most to the observed decline in infection risk at higher exposures. Another complicating factor is that body burdens of PCDD/Fs are strongly correlated with body burdens of mono- and di-*ortho *PCBs among the Uppsala mothers [20], making it even more difficult to draw conclusions about the contribution of non-dioxin-like and dioxin-like compounds to the suggested effects.

We did not study mechanisms behind the PCB and *p,p'*-DDE modulation of infection risks, and the study was not designed to study immunoactivation or -suppression. It may however be speculated that low-dose dioxin-like effects could be involved in the seemingly protective effect of the strongly correlated mono- and di-*ortho *PCBs, and *p,p'*-DDE. Studies of TCDD-exposed rodents have indicated that parts of the immune system may be activated at low exposure levels and suppressed at higher exposures [39-41]. Moreover, pre- and postnatal exposure of rats to low doses of TCDD enhanced innate immune response to influenza virus challenge, as indicated by neutrophilia and increased interferon γ levels in the lungs in both males and females, and suppressed cell mediated and antibody responses in females [42,43].

High prenatal CB 28+52+101 exposure increased the infection risk among the infants. Correlations between infant exposures to CB 28+52+101 and the other organochlorine compound groups studied by us were weak, probably due to different sources of exposure. Food is the major source of mono- and di-*ortho *PCB and *p,p'*-DDE exposure [44]. Our results suggest that food exposure to CB 28+52+101 generally is low, as indicated by the low levels of the compounds in serum lipid and mother's milk among a large part of the mothers. The high body burdens of CB 28+52+101 among some of the mothers, may be due to exposure in the in-door environment from PCB-laden building materials [30]. The associations between high exposure to CB 28+52+101 and increased risks of infections could reflect other exposures common for "PCB-houses". In our cohort, however, no associations were found between elevated concentrations of these three PCB congeners in maternal serum and residence during pregnancy in buildings built during the PCB usage period (1956–1972) [19]. It can nevertheless not be excluded that exposure to unknown environmental factors in PCB buildings contributed to the observed associations between PCB 28+52+101 exposure and risk of respiratory infections among the infants. No previous study has reported associations between infection risks among infants and pre- and postnatal exposure to CB-28, CB-52 and CB-101. No difference in health complaints was observed between children from a CB 28+52+101-contaminated school and children from a non-contaminated school [31].

The congeners CB-28, CB-52, CB-101, as well as the di-*ortho *PCBs, are non-dioxin-like PCBs. They may act through pathways independent of the aryl hydrocarbon receptor, which binds dioxin-like compounds [45-48]. Little is known about immunotoxic effects of non-dioxin-like PCB congeners CB-28, CB-52 and CB-101. In contrast to the dioxin-like CB-77, CB-126 and CB -69, CB-52 induced apoptosis in mice spleen cells *in vitro *[48]. Moreover, CB-28 and CB-52 caused rapid cell death among rat thymocytes *in vitro*, whereas CB-77 did not cause this effect at the same exposure concentration [49].

Only a few earlier studies have looked at associations between background PCB and *p,p'*-DDE exposure early in life and infant risk of respiratory infections. Among 3–6 months old Dutch and Inuit infants no significant associations were found between risks of respiratory infections and pre- or postnatal exposure to the congeners CB-118, CB-138, CB-153 and CB-180 [12,13,50]. In one of the Inuit studies a significant positive trend was found between prenatal PCB exposure and all infections, including respiratory and gastrointestinal tract infections and otitis media [50]. No significant trends of decreased or increase risk of respiratory infections with increased *p,p'*-DDE exposure were found among the Inuit infants [13,50].

It is difficult to determine the reasons behind the diverging results between studies. Pre-and postnatal exposure levels were higher among the Dutch and Inuit infants than among infants from Uppsala. For instance, the mean level of the sum of di-*ortho *congeners CB-138, CB-153 and CB-180 was 375–620 ng/g lipid in plasma/milk from Dutch and Inuit mothers (assuming a lipid content of 0.6% of blood plasma) [12,13], whereas it was 143 ng/g lipid in blood serum among the Uppsala women. Mean level of the PCB marker congener CB-153 in cord blood/mother's plasma lipids in the other Inuit study was 2-fold higher than the mean level found in mother's serum lipids in the Uppsala cohort [19,50]. Mean body burdens of *p,p'*-DDE among Inuit mothers were 3- to 11-fold higher than among the Uppsala mothers [13,50]. Moreover, the results in the Inuit studies may have been influenced by the general health status of the infants, since acute otitis media was common among the Inuit infants [13].

Our study is small and the results should therefore be interpreted with caution. It may have been difficult for mothers to correctly report infant health history of infants. Bias could have been introduced in the diagnosis of disease, since the disease diagnosis was not confirmed from medical records. The women did not get information about their body burdens of organochlorine compounds, avoiding bias in the reporting of diseases due to knowledge about the degree of exposure of the infant. Even though the results were adjusted with several potential confounders, unknown factors may still be involved in the associations observed. The results therefore have to be considered as hypothesis generating.

WBC and lymphocyte subset analyses were performed at the end of the 3 month study period. Therefore some of the observed associations with organochlorine compound exposure may be a consequence of the respiratory infections the infants had experienced during the study period. An increased number of lymphocytes and monocytes, as observed among infants with the highest CB 28+52+101 prenatal exposure, are indicators of infections and inflammation [51,52]. Therefore increased WBC counts could be related to more recent infections. However, infants that had an infection within 7 days before blood sampling for WBC and lymphocyte subset analyses were excluded from the statistical analyses. Moreover, adjustment of the immune cell results for infant's history of respiratory infections during the study period (yes/no) did not alter the observed associations. The immune system is very complex and we only studied a few immune markers. It is consequently difficult to draw conclusions about the relation between immune cell and infection results. The few observed shifts in immune cell numbers/percentages associated with organochlorine compound exposure were generally within the normal range [53,54], making it difficult to determine the clinical consequences of the observed shifts.

Prenatal mono- and di-*ortho *PCBs (including CB-153) exposures were negatively associated with the percentage of CD8^+ ^cytotoxic T-cells in the univariate analysis. Both animal and human studies suggest that early life exposure to dioxin-like compounds may modulate the numbers/percentages of CD8^+ ^cytotoxic T-cells. In mice and rat offspring, the population of CD8^+ ^cytotoxic T-cells was increased after pre- and postnatal TCDD exposure [2,55,56]. Among 18 month old Dutch infants a positive association was found between CD8^+ ^cell numbers and prenatal exposure to PCBs and PCDD/Fs in univariate analyses. No association was however found at 3 months of age [12]. The Dutch study was smaller than our study, and the statistically significant associations in the univariate analyses were not adjusted for potential confounders. In our study the negative associations between mono- and di-*ortho *PCB exposures and percentage of CD8+ were not statistically significant after adjustment for potential confounders. Differences in exposure levels of dioxin-like PCBs and PCDD/Fs also contribute to the difficulties to compare results between Uppsala (mean:19 pg total TEQ/g mother's milk lipid) and Dutch infants (64 pg TEQ/g lipid) [12,20].

We found no indications of influence of pre- and postnatal *p,p'*-DDE exposure on numbers of different types of lymphocytes, which is in accordance with the results reported for Inuit infants [13]. A negative association between prenatal *p,p'*-DDE exposure and percentage of eosinophilic granulocytes was however observed among the Uppsala infants. A study on German children in the ages 7–10 years reported a reduced eosinophilic granula content of eosinophilic granulocytes among children with the highest body burdens of *p,p'*-DDE [57]. Taken together the results indicate that eosinophilic granulocytes may respond to background exposures to *p,p'*-DDE among infants and children.

The WBC count and the analysis of lymphocyte subsets were performed on only 50–80 infants, and there were proportionally more girls in this subgroup than in the group of mother/infant pairs that did not donate blood. Moreover, fewer of the infants in the WBC group had been vaccinated. The immune cell results can therefore not be directly extrapolated to the whole study group. The immune cell numbers and percentages were only measured at one time point at the end of the 3 month study period, and we do not know if the results are representative for the other parts of the study period. Many statistical comparisons were made and it can therefore not be excluded that the results were due to chance. We used a strict significance level (p ≤ 0.01) and the results did not change significantly after adjustment for potential confounders, which reduces the possibility of chance findings.

## Conclusion

This hypothesis generating study suggest that background exposure to PCBs and *p,p'*-DDE early in life modulate immune system development. Strong correlations between mono- and di-*ortho *PCBs (including CB-153), and *p,p'*-DDE exposures make it difficult to identify the most important contributor to the suggested immunomodulation, and to separate effects due to pre- and postnatal exposure. Our findings may have consequences for the health development during childhood, since respiratory infections early in life may be risk factors for asthma and middle ear infections.

## Abbreviations

CB: chlorinated biphenyl; CD: clusters of differentiation; CD3^+^: T-lymphocytes; CD4^+^: T-helper cells; CD8^+^: cytotoxic cells; CD19^+^: B-lymphocytes; CD56^+^: natural killer cells; CI: confidence interval; DDE: dichlorodiphenylchloroethane; IUPAC: International Union of Pure and Applied Chemistry; PCB: polychlorinated biphenyl; SD: standard deviation; SE: standard error; WBC: white blood cell.

## Competing interests

The authors declare that they have no competing interests.

## Authors' contributions

AG participated in the planning of the study, data collection and data analyses, and wrote the first draft of the manuscript. AT participated in the planning of the study, data collection and data analyses. MA participated in the planning of the study and was responsible for the organochlorine compound analysis. AJ participated in the planning of the study and data analyses, and was responsible for the lymphocyte subset analyses. POD participated in the planning of the study and data collection. GR was responsible for the WBC analyses. SC participated in the planning of the study and data collection. All authors participated in the preparation of the final manuscript and approved the submission.

## Supplementary Material

Additional file 1 **Respiratory infections and white blood cell and lymphocyte subset numbers/percentages in 3 months old infants exposed to PCB and *p,p'*-DDE postnatally.** Unadjusted means (± SE) of white blood cell numbers (x10^9^) in 3-month-old infants exposed to organochlorines postnatally. Click here for file

Additional file 2 **Respiratory infections and white blood cell and lymphocyte subset numbers/percentages in 3 months old infants exposed to PCB and *p,p'*-DDE postnatally.** Unadjusted and adjusted means (± SE) of white blood cell percentages in 3-month-old infants exposed to organochlorines postnatally. Click here for file

Additional file 3 **Respiratory infections and white blood cell and lymphocyte subset numbers/percentages in 3 months old infants exposed to PCB and *p,p'*-DDE postnatally.** Unadjusted and adjusted means (± SE) of lymphocyte subset numbers (x10^9^) in 3-month-old infants exposed to organochlorines postnatally. Click here for file

Additional file 4 **Respiratory infections and white blood cell and lymphocyte subset numbers/percentages in 3 months old infants exposed to PCB and *p,p'*-DDE postnatally.** Unadjusted and adjusted means (± SE) of lymphocyte subset percentages in 3-month-old infants exposed to organochlorines postnatally. Click here for file

Additional file 5 **Respiratory infections and white blood cell and lymphocyte subset numbers/percentages in 3 months old infants exposed to PCB and *p,p'*-DDE postnatally.** Odds ratios (95% CI) for associations between respiratory infections, lasting 3 days or more during the first 3 months after birth, and pre- or postnatal exposure to organochlorine compounds. Click here for file

## References

[B1] Banerjee BD (1999). The influence of various factors on immune toxicity assessment of pesticide chemicals. Toxicol Lett.

[B2] Gehrs BC, Riddle MM, Williams WC, Smialowicz RJ (1997). Alterations in the developing immune system of the F344 rat after perinatal exposure to 2,3,7,8-tetrachlorodibenzo-p-dioxin: II. Effects on the pup and the adult. Toxicology.

[B3] Kerkvliet NI, Baecher-Steppan L, Smith BB, Youngberg JA, Henderson MC, Buhler DR (1990). Role of the Ah locus in suppression of cytotoxic T lymphocyte activity by halogenated aromatic hydrocarbons (PCBs and TCDD): structure-activity relationships and effects in C57Bl/6 mice congenic at the Ah locus. Fundam Appl Toxicol.

[B4] Guo YL, Lambert GH, Hsu CC, Hsu MM (2004). Yucheng: health effects of prenatal exposure to polychlorinated biphenyls and dibenzofurans. Int Arch Occup Environ Health.

[B5] Yu ML, Hsin JW, Hsu CC, Chan WC, Guo YL (1998). The immunologic evaluation of the Yucheng children. Chemosphere.

[B6] SCF (2000). Opinion of the SCF on the risk assessment of dioxins and dioxin-like PCBs in food. Opinion of the Scientific Committee on Foods.

[B7] JECFA (2002). Safety evaluation of certain food additives and contaminants. Polychlorinated dibenzodioxins, polychlorinated dibenzofurans, and coplanar polychlorinated biphenyls. IPCS INCHEM Report.

[B8] Belles-Isles M, Ayotte P, Dewailly E, Weber JP, Roy R (2002). Cord blood lymphocyte functions in newborns from a remote maritime population exposed to organochlorines and methylmercury. J Toxicol Environ Health A.

[B9] Nagayama J, Tsuji H, Iida T, Hirakawa H, Matsueda T, Okamura K, Hasegawa m, Sato K, Ma HY, Yanagawa T, Igarashi H, Fukushige J, Watanabe T (1998). Postnatal exposure to chlorinated dioxins and related chemicals on lymphocyte subsets in Japanese breast-fed infants. Chemosphere.

[B10] ten Tusscher GW, Steerenberg PA, van Loveren H, Vos JG, Borne AE von dem, Westra M, Slikke JW van der, Olie K, Pluim HJ, Koppe JG (2003). Persistent hematologic and immunologic disturbances in 8-year-old Dutch children associated with perinatal dioxin exposure. Environ Health Perspect.

[B11] Weisglas-Kuperus N, Patandin S, Berbers GA, Sas TC, Mulder PG, Sauer PJ, Hooijkaas H (2000). Immunologic effects of background exposure to polychlorinated biphenyls and dioxins in Dutch preschool children. Environ Health Perspect.

[B12] Weisglas-Kuperus N, Sas TC, Koopman-Esseboom C, Zwan CW van der, De Ridder MA, Beishuizen A, Hooijkaas H, Sauer JP (1995). Immunologic effects of background prenatal and postnatal exposure to dioxins and polychlorinated biphenyls in Dutch infants. Pediatr Res.

[B13] Dewailly E, Ayotte P, Bruneau S, Gingras S, Belles-Isles M, Roy R (2000). Susceptibility to infections and immune status in Inuit infants exposed to organochlorines. Environ Health Perspect.

[B14] Park H-Y, Hertz-Picciotto I, Petrik J, Palkovicova L, Kocan A, Trnovec T (2008). Prenatal PCB exposure and thymus size at birth in neonates in eastern Slovakia. Environ Health Perspect.

[B15] Weisglas-Kuperus N, Vreugdenhil HJ, Mulder PG (2004). Immunological effects of environmental exposure to polychlorinated biphenyls and dioxins in Dutch school children. Toxicol Lett.

[B16] Karmaus W, Kuehr J, Kruse H (2001). Infections and atopic disorders in childhood and organochlorine exposure. Arch Environ Health.

[B17] Sunyer J, Torrent M, Munoz-Ortiz L, Ribas-Fito N, Carrizo D, Grimalt J, Anto JM, Cullinin P (2005). Prenatal dichlorodiphenyldichloroethylene (DDE) and asthma in children. Environ Health Perspect.

[B18] Heilmann C, Grandjean P, Weihe P, Nielsen F, Budtz-Jorgensen E (2006). Reduced antibody responses to vaccinations in children exposed to polychlorinated biphenyls. PLoS Med.

[B19] Glynn A, Aune M, Darnerud PO, Cnattingius S, Bjerselius R, Becker W, Lignell S (2007). Determinants of serum concentrations of organochlorine compounds in Swedish pregnant women: a cross-sectional study. Environ Health.

[B20] Glynn AW, Atuma S, Aune M, Darnerud PO, Cnattingius S (2001). Polychlorinated biphenyl congeners as markers of toxic equivalents of polychlorinated biphenyls, dibenzo-p-dioxins and dibenzofurans in breast milk. Environ Res.

[B21] Clausson B, Granath F, Ekbom A, Lundgren S, Nordmark A, Signorello LB, Cnattingius S (2002). Effect of caffeine exposure during pregnancy on birth weight and gestational age. Am J Epidemiol.

[B22] Graske A, Thuvander A, Johannisson A, Gadhasson I, Schutz A, Festin R, Glynn AW (2000). Influence of aluminium on the immune system – an experimental study on volunteers. Biometals.

[B23] Ayotte P, Muckle G, Jacobson JL, Jacobson SW, Dewailly E (2003). Assessment of pre- and postnatal exposure to polychlorinated biphenyls: lessons from the Inuit Cohort Study. Environ Health Perspect.

[B24] Covaci A, Jorens P, Jacquemyn Y, Schepens P (2002). Distribution of PCBs and organochlorine pesticides in umbilical cord and maternal serum. Sci Total Environ.

[B25] Jaraczewska K, Lulek J, Covaci A, Voorspoels S, Kaluba-Skotarczak A, Drews K, Schepens P (2006). Distribution of polychlorinated biphenyls, organochlorine pesticides and polybrominated diphenyl ethers in human umbilical cord serum, maternal serum and milk from Wielkopolska region, Poland. Sci Total Environ.

[B26] Lanting CI, Fidler V, Huisman M, Boersma ER (1998). Determinants of polychlorinated biphenyl levels in plasma from 42-month-old children. Arch Environ Contam Toxicol.

[B27] LaKind JS, Berlin CM, Park CN, Naiman DQ, Gudka NJ (2000). Methodology for characterizing distributions of incremental body burdens of 2,3,7,8-TCDD and DDE from breast milk in North America nursing women. J Toxicol Environ Health A.

[B28] Gabrio T, Piechotowski I, Wallenhorst T, Klett M, Cott L, Friebel P, Link B, Schwenk M (2000). PCB-blood levels in teachers, working in PCB-contaminated schools. Chemosphere.

[B29] Johansson N, Hanberg A, Bergek S, Tysklind M (2001). PCB in sealant is influencing the levels in indoor air. Organohalogen Compounds.

[B30] Johansson N, Hanberg A, Wingfors H, Tysklind M (2003). PCB in building sealant is influencing PCB levels in blood of residents. Organohalogen Compounds.

[B31] Liebl B, Schettgen T, Kerscher G, Broding HC, Otto A, Angerer J, Drexel H (2004). Evidence for increased internal exposure to lower chlorinated polychlorinated biphenyls (PCB) in pupils attending a contaminated school. Int J Hyg Environ Health.

[B32] Berg M Van den, Birnbaum L, Bosveld AT, Brunstrom B, Cook P, Feeley M, Giesy JP, Hanberg A, Hasegaw R, Kennedy SW, Kubiak T, Larsen JC, Van Leeuwen FX, Liem AK, Nolt C, Peterson RE, Poellinger L, Safe S, Schrenk D, Tillitt D, Tysklind M, Younes M, Waern F, Zacharewski T (1998). Toxic equivalency factors (TEFs) for PCBs, PCDDs, PCDFs for humans and wildlife. Environ Health Perspect.

[B33] Bjorksten B (2005). Genetic and environmental risk factors for the development of food allergy. Curr Opin Allergy Clin Immunol.

[B34] Linneberg A, Petersen J, Gronbaek M, Benn CS (2004). Alcohol during pregnancy and atopic dermatitis in the offspring. Clin Exp Allergy.

[B35] Rottem M, Shoenfeld Y (2004). Vaccination and allergy. Curr Opin Otolaryngol Head Neck Surg.

[B36] Braback L, Bjor O, Nordahl G (2003). Early determinants of first hospital admissions for asthma and acute bronchitis among Swedish children. Acta Paediatr.

[B37] van Odijk J, Kull I, Borres MP, Brandtzaeg P, Edberg U, Hanson LA, Host A, Kuitunen M, Olsen SF, Skerfving S, Sundell J, Wille S (2003). Breastfeeding and allergic disease: a multidisciplinary review of the literature (1966–2001) on the mode of early feeding in infancy and its impact on later atopic manifestations. Allergy.

[B38] Kulig M, Luck W, Lau S, Niggemann B, Bergmann R, Klettke U, Guggenmoos-Holzmann I, Wahn U (1999). Effect of pre- and postnatal tobacco smoke exposure on specific sensitization to food and inhalant allergens during the first 3 years of life. Multicenter Allergy Study Group, Germany. Allergy.

[B39] Fan F, Wierda D, Rozman KK (1996). Effects of 2,3,7,8-tetrachlorodibenzo-p-dioxin on humoral and cell-mediated immunity in Sprague-Dawley rats. Toxicology.

[B40] Fan F, Yan B, Wood G, Viluksela M, Rozman KK (1997). Cytokines (IL-1beta and TNFalpha) in relation to biochemical and immunological effects of 2,3,7,8-tetrachlorodibenzo-p-dioxin (TCDD) in rats. Toxicology.

[B41] Neubert R, Golor G, Stahlmann R, Helge H, Neubert D (1992). Polyhalogenated dibenzo-p-dioxins and dibenzofurans and the immune system. 4. Effects of multiple-dose treatment with 2,3,7,8-tetrachlorodibenzo-p-dioxin (TCDD) on peripheral lymphocyte subpopulations of a non-human primate (Callithrix jacchus). Arch Toxicol.

[B42] Vorderstrasse B, Cundiff JA, Lawrence BP (2004). Potent aryl hydrocarbon receptor agonist 2,3,7,8-tetrachlorodibenzo-p-dioxin impairs the cell-mediated immune response to infection with Influenza A virus, but enhances elements of innate immunity. J Immunotoxicol.

[B43] Vorderstrasse BA, Cundiff JA, Lawrence BP (2006). A dose-response study of the effects of pre-natal and lactational exposure to TCDD on the immune response to Influenza A virus. J Toxicol Environ Health A.

[B44] Ankarberg E, Aune M, Concha G, Darnerud PO, Glynn A, Lignell S, Törnkvist A (2007). Risk assessment of persistent chlorinated and brominated environmental pollutants in food. NFA Report 9/07.

[B45] Hogaboam JP, Moore AJ, Lawrence BP (2008). The aryl hydrocarbon receptor affects distinct tissue compartments during ontogeny of the immune system. Toxicol Sci.

[B46] Teske S, Bohn AA, Hogaboam JP, Lawrence BP (2008). Aryl hydrocarbon receptor targets pathways extrinsic to bone marrow cells to enhance neutrophil recruitment during influenza infection. Toxicol Sci.

[B47] Levin M, Morsey B, Mori C, Nambiar PR, De Guise S (2005). Non-coplanar PCB-mediated modulation of human leukocyte phagocytosis: a new mechanism for immunotoxicity. J Toxicol Environ Health A.

[B48] Jeon YJ, Youk EO, Lee SH, Suh J, Na YJ, Kim HM (2002). Polychlorinated biphenyl-induced apopotosis of murine spleen cells is aryl hydrocarbon receptor independent but caspases dependent. Toxicol Appl Pharmacol.

[B49] Tan Y, Song R, Lawrence D, Carpenter DO (2004). *Ortho*-substituted but not coplanar PCBs rapidly kill cerebellar granule cells. Toxicol Sci.

[B50] Dallaire F, Dewailly E, Muckle G, Vézina C, Jacobson SW, Jacobson JL, Ayotte P (2004). Acute infections and environmental exposure to organochlorines in Inuit infants from Nunavik. Environ Health Perspect.

[B51] Jakubik LD, Cockerham J, Altmann AR, Grossman MB (2003). The ABCs of pediatric laboratory interpretation: Understanding the CBC with Differential and LFTs. Pediatr Nurs.

[B52] George-Gay B, Parker K (2003). Understanding the complete blood count with differential. J PeriAnesth Nurs.

[B53] Bellamy GJ, Hinchliffe RF, Crawshaw KC, Finn A, Bell F (2000). Total and differential leucocyte counts in infants at 2, 5 and 13 months of age. Clin Lab Haematol.

[B54] Shearer WT, Rosenblatt HM, Gelman RS, Oyomopito R, Plaeger S, Stiehm ER, Wara DW, Douglas SD, Luzuriaga K, McFarland EJ, Yogev R, Rathore MH, Levy W, Graham BL, Spector SA (2003). Lymphocyte subsets in healthy children from birth through 18 years of age: the Pediatric AIDS Clinical Trials Group P1009 study. J Allergy Clin Immunol.

[B55] Gehrs BC, Smialowicz RJ (1997). Alterations in the developing immune system of the F344 rat after perinatal exposure to 2,3,7,8-tetrachlorodibenzo-p-dioxin I. [correction of II]. Effects on the fetus and the neonate. Toxicology.

[B56] Holladay SD, Lindstrom P, Blaylock BL, Comment CE, Germolec DR, Heindell JJ, Luster MI (1991). Perinatal thymocyte antigen expression and postnatal immune development altered by gestational exposure to tetrachlorodibenzo- p-dioxin (TCDD). Teratology.

[B57] Karmaus W, Brooks KR, Nebe T, Witten J, Obi-Osius N, Kruse H (2005). Immune function biomarkers in children exposed to lead and organochlorine compounds: a cross-sectional study. Environ Health.

