# Supplementary material for: Immune cell counts and risks of respiratory infections among infants exposed pre- and postnatally to organochlorine compounds: a prospective study
Source: Environ Health. 2008 Dec 4;7:62. doi: 10.1186/1476-069X-7-62 (PMC2637846; doi:10.1186/1476-069X-7-62)
Supplement: Additional file 1 — Respiratory infections and white blood cell and lymphocyte subset numbers/percentages in 3 months old infants exposed to PCB and p,p'-DDE postnatally. Unadjusted means (± SE) of white blood cell numbers (x109) in 3-month-old infants exposed to organochlorines postnatally. [file 1476-069X-7-62-S1.pdf]

Table A. Unadjusted means (±SE) of white blood cell numbers ($x10^9$/L) in 3 month old infants exposed to organochlorines postnatally[a]

| | White blood cells | Neutrophils | Eosinophils | Lymphocytes | Monocytes |
|---|---|---|---|---|---|
| CB 28+52+101 postnatal | | | | | |
| 0-5.5 ng/g fw*days | 7.65±0.39 | 1.66±0.13 | 0.32±0.03 | 5.13±0.31 | 0.35±0.04 |
| 5.6-11.8 | 8.21±0.40 | 1.82±0.13 | 0.32±0.03 | 5.52±0.32 | 0.34±0.04 |
| 11.9-76.3 | 8.67±0.41 | 1.49±0.14 | 0.28±0.03 | 6.00±0.33 | 0.40±0.04 |
| CB-153 postnatal | | | | | |
| 0-120 ng/g fw*days | 8.30±0.41 | 1.78±0.18 | 0.32±0.03 | 5.55±0.32 | 0.36±0.04 |
| 121-209 | 8.09±0.41 | 1.98±0.18 | 0.30±0.03 | 5.34±0.33 | 0.35±0.04 |
| 210-396 | 8.10±0.41 | 1.54±0.18 | 0.29±0.03 | 5.74±0.33 | 0.37±0.04 |
| Di-*ortho* PCB postnatal | | | | | |
| 0-267 ng/g fw*days | 8.33±0.39 | 1.63±0.14 | 0.33±0.03 | 5.63±0.31 | 0.37±0.04 |
| 268-412 | 8.11±0.41 | 1.83±0.14 | 0.29±0.03 | 5.29±0.33 | 0.36±0.04 |
| 413-830 | 8.02±0.43 | 1.48±0.14 | 0.30±0.03 | 5.70±0.35 | 0.36±0.04 |
| Mono-*ortho* PCB postnatal | | | | | |
| 0-8.2 pg TEQ/g fw*days | 8.13±0.39 | 1.65±0.13 | 0.34±0.03 | 5.58±0.30 | 0.36±0.04 |
| 8.3-12.1 | 8.28±0.40 | 1.85±0.14 | 0.30±0.03 | 5.26±0.32 | 0.37±0.04 |
| 12.2-26.0 | 8.07±0.42 | 1.43±0.15 | 0.26±0.03 | 5.84±0.36 | 0.34±0.04 |
| *p,p´*-DDE postnatal | | | | | |
| 0-211 ng/g fw*days | 8.18±0.39 | 1.80±0.13 | 0.35±0.03 | 5.41±0.30 | 0.37±0.04 |
| 212-413 | 8.00±0.42 | 1.63±0.15 | 0.31±0.03 | 5.23±0.33 | 0.34±0.04 |
| 414-2199 | 8.32±0.43 | 1.49±0.14 | 0.24±0.03 | 6.05±0.34 | 0.37±0.04 |

[a] Infants with an ongoing infection at the time of sampling were excluded, as well as infants that had an infection within 7 days before sampling. Postnatal exposure: breast milk levels (ng or pg/g fresh weight)*days of nursing*(%of full nursing/100). CB 28+52+101=CB-28, CB-52, CB-101; Di-*ortho* PCB=CB-138, CB-153, CB-180; Mono-*ortho* PCB TEQ=CB-105, CB-118, CB-156, CB-167 [32]. N=74-75.

*$p \leq 0.01$
