# Supplementary material for: Immune cell counts and risks of respiratory infections among infants exposed pre- and postnatally to organochlorine compounds: a prospective study
Source: Environ Health. 2008 Dec 4;7:62. doi: 10.1186/1476-069X-7-62 (PMC2637846; doi:10.1186/1476-069X-7-62)
Supplement: Additional file 2 — Respiratory infections and white blood cell and lymphocyte subset numbers/percentages in 3 months old infants exposed to PCB and p,p'-DDE postnatally. Unadjusted and adjusted means (± SE) of white blood cell percentages in 3-month-old infants exposed to organochlorines postnatally. [file 1476-069X-7-62-S2.pdf]

Table B. Unadjusted and adjusted means (±SE) of white blood cell percentages in 3 month old infants exposed to organochlorines postnatally[a]

	Neutrophils	Eosinophils	Lymphocytes	Monocytes
CB 28+52+101 postnatal				
0-5.5 ng/g fw*days	23.22±1.74	4.56±0.42	66.54±1.75	4.63±0.40
5.6-11.8	24.13±1.82	4.23±0.44	68.00±1.87	4.02±0.40
11.9-76.3	21.70±1.82	3.42±0.44	70.72±1.87	4.49±0.41
CB-153 postnatal				
0-120 ng/g fw*days	24.62±1.75	4.23±0.44	66.86±1.83	4.01±0.41
121-209	24.11±1.75	4.18±0.44	67.61±1.83	4.53±0.40
210-396	20.23±1.78	3.83±0.45	70.55±1.83	4.59±0.40
Di-*ortho* PCB postnatal				
0-267 ng/g fw*days	23.86±1.66	4.23±0.42	67.57±1.74	3.71±0.44
268-412	25.10±1.69	4.10±0.43	66.64±1.79	4.62±0.39
413-830	19.35±1.89	3.88±0.48	71.34±1.95	4.90±0.50
Mono-*ortho* PCB postnatal				
0-8.2 pg TEQ/g fw*days	23.48±1.63	4.52±0.42	66.92±1.68	3.84±0.44
8.3-12.1	25.78±1.67	4.06±0.43	66.56±1.79	4.82±0.39
12.2-26.0	19.00±1.86	3.55±0.48	72.24±1.92	4.50±0.47
p,p´-DDE postnatal				
0-211 ng/g fw*days	25.23±1.61	4.62±0.40	65.77±1.66	4.17±0.40
212-413	23.95±1.75	4.30±0.43	67.50±1.81	4.38±0.41
414-2199	19.18±1.83	3.17±0.45	72.41±1.84*	4.64±0.45
0-211 ng/g fw*days[b]			62.81±2.59	
212-413			65.66±2.70	
414-2199			70.87±2.99	

[a] Infants with an ongoing infection at the time of sampling were excluded, as well as infants that had an infection within 7 days before sampling. Postnatal exposure: breast milk levels (ng or pg/g fresh weight)*days of nursing*(%of full nursing/100). CB 28+52+101=CB-28, CB-52, CB-101; Di-*ortho* PCB=CB-138, CB-153, CB-180; Mono-*ortho* PCB TEQ=CB-105, CB-118, CB-156, CB-167 [32]. N=79.

[b]Infants in the highest exposure group had a significantly higher percentage of lymphocytes than infants with the lowest exposure in univariate analysis. Therefore means were adjusted for age of the mother, smoking and alcohol consumption during pregnancy, mother's education, vaccination of the infant, nursing of the infant, age of the infant, and infant´s history of respiratory infections.

*$p \leq 0.01$
