# Supplementary material for: Immune cell counts and risks of respiratory infections among infants exposed pre- and postnatally to organochlorine compounds: a prospective study
Source: Environ Health. 2008 Dec 4;7:62. doi: 10.1186/1476-069X-7-62 (PMC2637846; doi:10.1186/1476-069X-7-62)
Supplement: Additional file 3 — Respiratory infections and white blood cell and lymphocyte subset numbers/percentages in 3 months old infants exposed to PCB and p,p'-DDE postnatally. Unadjusted and adjusted means (± SE) of lymphocyte subset numbers (x109) in 3-month-old infants exposed to organochlorines postnatally. [file 1476-069X-7-62-S3.pdf]

Table C. Unadjusted and adjusted means (±SE) of lymphocyte subset numbers (x10^9/L) in 3 month old infants exposed to organochlorines postnatally[a]

	$CD19^+$	$CD3^+$	$CD4^+CD8^-$	$CD4^-CD8^+$	$CD56^+$
CB 28+52+101 postnatal					
0-5.5 ng/g fw*days	0.82±0.12	3.77±0.32	3.13±0.26	0.68±0.06	0.06±0.01
5.6-11.8	0.83±0.13	4.16±0.35	3.26±0.28	0.74±0.07	0.05±0.01
11.9-76.3	1.00±0.12	3.66±0.34	2.88±0.27	0.68±0.06	0.05±0.01
CB-153 postnatal					
0-134 ng/g fw*days	0.76±0.12	3.92±0.33	3.20±0.27	0.76±0.06	0.04±0.01
135-217	1.00±0.12	3.78±0.33	3.06±0.26	0.72±0.06	0.07±0.01*
218-396	0.89±0.13	9.97±0.37	3.11±0.29	0.62±0.07	0.05±0.01
0-134 ng/g fw*days[b]					0.04±0.01
135-217					0.07±0.01*
218-396					0.06±0.01
Di-*ortho* PCB postnatal					
0-267 ng/g fw*days	0.80±0.11	3.90±0.31	3.22±0.25	0.73±0.06	0.04±0.01
268-412	0.98±0.12	3.61±0.34	2.86±0.27	0.74±0.06	0.06±0.01
413-830	0.87±0.13	4.07±0.37	3.18±0.29	0.60±0.07	0.06±0.01
Mono-*ortho* PCB postnatal					
0-8.2 pg/g fw*days	0.85±0.12	4.01±0.31	3.28±0.25	0.75±0.06	0.05±0.01
8.3-12.1	0.90±0.12	3.62±0.34	2.95±0.27	0.74±0.06	0.05±0.01
12.2-26.0	0.91±0.13	3.89±0.37	2.99±0.29	0.57±0.07	0.06±0.01
p,p´-DDE postnatal					
0-211 ng/g fw*days	0.80±0.12	3.79±0.31	2.99±0.25	0.71±0.06	0.04±0.01
212-413	0.97±0.13	3.61±0.35	3.00±0.28	0.72±0.06	0.06±0.01
414-2199	0.90±0.13	4.17±0.35	3.29±0.28	0.67±0.07	0.06±0.01

[a] Infants with an ongoing infection at the time of sampling were excluded, as well as infants that had an infection within 7 days before sampling. Postnatal exposure: breast milk levels (ng or pg/g fresh weight)*days of nursing*(%of full nursing/100). CB 28+52+101=CB-28, CB-52, CB-101; Di-*ortho* PCB=CB-138, CB-153, CB-180; Mono-*ortho* PCB TEQ=CB-105, CB-118, CB-156, CB-167 [32]. N=45.

[b]Infants in the second highest exposure group had a significantly higher number of CD56+ lymphocytes than infants with the lowest exposure in univariate analysis. Therefore means were adjusted for age of the mother, smoking and alcohol consumption during pregnancy, mother's education, vaccination of the infant, nursing of the infant, age of the infant, and infant´s history of respiratory infections.

*p<0.01
