# Supplementary material for: Immune cell counts and risks of respiratory infections among infants exposed pre- and postnatally to organochlorine compounds: a prospective study
Source: Environ Health. 2008 Dec 4;7:62. doi: 10.1186/1476-069X-7-62 (PMC2637846; doi:10.1186/1476-069X-7-62)
Supplement: Additional file 5 — Respiratory infections and white blood cell and lymphocyte subset numbers/percentages in 3 months old infants exposed to PCB and p,p'-DDE postnatally. Odds ratios (95% CI) for associations between respiratory infections, lasting 3 days or more during the first 3 months after birth, and pre- or postnatal exposure to organochlorine compounds. [file 1476-069X-7-62-S5.pdf]

Table E. Odds ratios (95% CI) for associations between respiratory infections, lasting 3 days or more during the first 3 months after birth, and pre- or postnatal exposure to organochlorine compounds[a]

	Model[b]	Exposure categories[c]			
		Category 1	Category 2	Category 3	Category 4
**Prenatal**					
CB 28+52+101	Multivariate	1.0	2.1 (0.85-5.1)	3.3 (1.4-7.9)*	
CB-153	Multivariate	1.0	0.42 (0.15-1.2)	0.28 (0.09-0.85)*	0.43 (0.12-1.5)
Di-*ortho* PCB	Multivariate	1.0	0.28 (0.10-0.80)*	0.23 (0.07-0.73)*	0.32 (0.09-1.1)
Mono-*ortho* PCB TEQ	Multivariate	1.0	0.42 (0.15-1.2)	0.36 (0.11-1.2)	0.28 (0.07-1.1)
**Postnatal**					
CB-153	Multivariate	1.0	0.43 (0.14-1.3)	0.41 (0.13-1.3)	0.24 (0.07-0.86)*
Di-*ortho* PCB	Multivariate	1.0	0.27 (0.08-0.89)*	0.31 (0.10-1.0)	0.17 (0.05-0.61)*
Mono-*ortho* PCB TEQ	Multivariate	1.0	0.26 (0.08-0.88)*	0.46 (0.14-1.4)	0.33 (0.10-1.2)
p,p´-DDE	Multivariate	1.0	0.17 (0.05-0.60)*	0.48 (0.16-1.4)	0.64 (0.21-1.9)

[a]Prenatal exposure: Lipid adjusted mother´s serum organochlorine compound concentrations in late pregnancy (week 32-34). Postnatal exposure: mother´s milk concentrations on a fresh weight basis*days of nursing*(%of full nursing/100). Di-*ortho* PCB=CB-138, CB-153, CB-180; Mono-*ortho* PCB TEQ=CB-105, CB-118, CB-156, CB-167 [32]. Prenatal exposure:N=190. Postnatal exposure:N=175. Statistical analyses were performed when statistically significant associations between organochlorine compound exposure and infection risk were found (see Table 5).

[b]Multivariate model included the independent variables age of the mother, smoking and alcohol during pregnancy, mother's education, vaccination of the infant, nursing of the infant, and age of the infant.

[c]Pre- and postnatal exposure was categorized with the lowest exposure in Category 1 and increasing exposure with increasing Category number. Efforts were made to have equal numbers of participants in the exposure categories for each compound group.

*Significantly different from the odds ratio of the reference category ($p \leq 0.05$).
